# Aorto-bronchial fistula masquerading as haematemesis: a rare late complication of thoracic aortic vascular grafts

**DOI:** 10.1093/jscr/rjad131

**Published:** 2023-03-14

**Authors:** Timothy Peacock, Alexandra M Limmer, Assad Zahid

**Affiliations:** Department of Colorectal Surgery, Liverpool Hospital, Sydney, NSW, Australia; Department of Colorectal Surgery, Liverpool Hospital, Sydney, NSW, Australia; Department of Colorectal Surgery, Liverpool Hospital, Sydney, NSW, Australia

**Keywords:** aorto-bronchial, fistula, haematemesis, vascular graft

## Abstract

Aorto-bronchial fistula is an exceedingly rare pathology with high mortality. Late vascular graft infection may occur secondary to haematogenous seeding of bacteria from a distant source such as gastrointestinal infection. We present an unusual case of aorto-bronchial fistula masquerading as haematemesis in a patient with sigmoid diverticulitis, and review the pathophysiology, diagnosis, surgical and endovascular management of aorto-bronchial fistulas.

## INTRODUCTION

Aorto-bronchial fistula is an exceedingly rare pathology with high mortality. We present an unusual case of aorto-bronchial fistula masquerading as haematemesis in a patient with sigmoid diverticulitis.

## CASE REPORT

**Figure 1:**
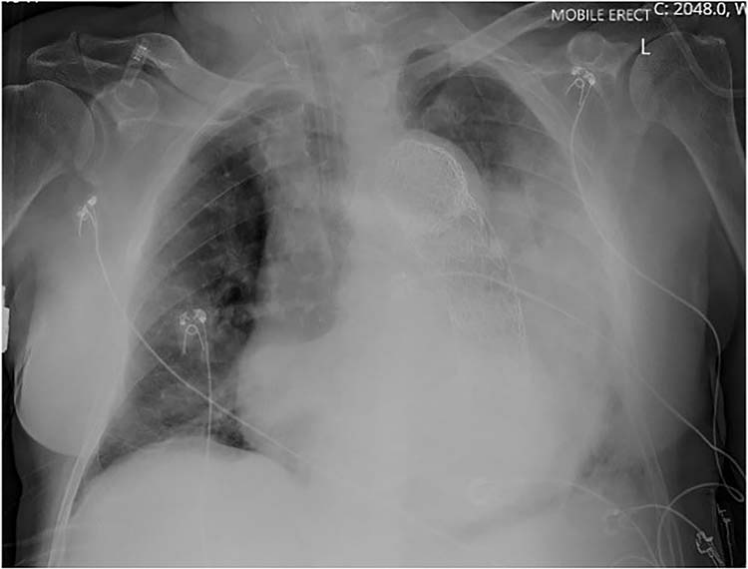
Erect AP chest X-ray indicating opacification of the left mid and lower zones, with thoracic vascular graft *in situ*.

**Figure 2:**
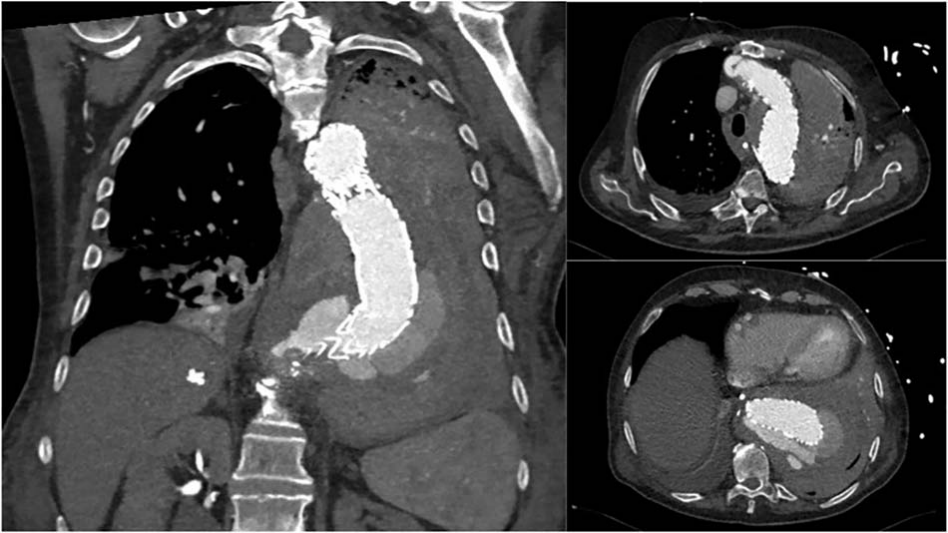
Coronal and axial CT thoracic aortogram indicating large Type 1 endoleak with large left haemothorax.

An 84-year-old male was admitted with sigmoid diverticulitis with a contained perforation in the colonic mesentery. His medical background included open thoracoabdominal aortic aneurysm repair 15 years prior, and chronic obstructive pulmonary disease. He was managed nonoperatively with intravenous antibiotics. On day 5 of admission, he acutely deteriorated with hypotension, tachypnoea and 200 mL of fresh haematemesis. Chest X-ray indicated opacification of the left lung, presumed to represent aspiration of haematemesis (Fig. 1). The patient received blood transfusion and proceeded to urgent gastroscopy, which identified old blood in the stomach and a Dieulafoy lesion, which was clipped. Post-procedure, the patient remained haemodynamically normal in intensive care, however had worsening opacification of his left lung on X-ray. The patient underwent bronchoscopy, with extraction of a further 200 mL of clot from the bronchial tree. In light of his vascular history, the patient proceeded to computed tomography (CT) angiography, which indicated a large Type 1 endoleak at the inferior margin of his thoracic aortic endograft, with a large left haemothorax and complete collapse of the left lung (Fig. 2). In the context of his comorbidities with active deterioration, the patient was palliated.

## DISCUSSION

Aorto-pleural fistulas have been described in the literature since 1914, with the first patient being affected by pulmonary tuberculosis [1]. In the 1960s, it was found that patients undergoing aortic arch replacements were at high risk of developing aorto-bronchial fistulas [2]. While most commonly occurring after repair of the descending thoracic aorta, fistulas have also been associated with repair of aortic coarctation, patent ductus arteriosus and tetralogy of Fallot; aortic and mitral valve replacement; traumatic rupture of the descending thoracic aorta; Takayasu arteritis and aortic sarcomas [3]. Before 1960, the most common cause of aortic aneurysms was mycotic aneurysms, with a shift now to postsurgical and degenerative aneurysms [3]. Operative factors leading to the formation of pseudoaneurysm include placing tight sutures, and silk suture fragmentation and dissolution in patients with Teflon grafts [4, 5]. Procedures to the bronchial tree can also cause development of fistulas including bronchial stenting with metal stents in patients after lung transplantation, paediatric bronchomalacia and tracheal resection [6–8].

There is also an association between vascular graft infection and fistula formation. Late vascular graft infection may occur secondary to haematogenous seeding of bacteria from a distant source such as gastrointestinal infection. Gram negative enteric organisms such as *Escherichia coli*, Klebsiella and Enterobacter in particular exhibit high virulence and cause vascular wall destruction. A study including 1258 patients with vascular graft infection found that Streptococcus and *E. coli* were the primary causative organisms of aortoenteric fistula formation [9].

The pathophysiology of aorto-bronchial fistula is poorly understood, but is likely to occur because of the extravasation of blood into the surroundings tissues [10]. The haematoma is initially contained within the vascular layers and fibrous tissue, resulting in development of a neointima. As the aneurysm enlarges around the graft, it causes progressive airway compression and localized pressure necrosis. Eventually, the lung undergoes chronic pulsatile erosion from the vascular mass [11]. Once the tension of the pseudoaneurysm becomes critical, it ruptures into the airway [10].

Most patients present with haemoptysis, which may range from a small herald bleed to large volume bleeding with exsanguination. The degree of haemorrhage on presentation depends on the underlying pathology, with small fistulas easily occluded by clots. These clots may readily be dislodged, resulting in further haemorrhage [3]. Often this process of clotting with ongoing haemorrhaging results in the fistula becoming larger in size, until there is massive exsanguination of blood. Other associated features may include dyspnoea, pulmonary rales, dysphonia, tachypnoea, hypoxia and haemodynamic instability from haemorrhagic shock [3].

Diagnosis can be definitively obtained via CT chest with arterial phase contrast. Unless there is active arterial haemorrhage at the time of scanning, the fistula itself may not be clearly demonstrated. However, ancillary features will be evident such as an aortic endoleak with associated lung consolidation or fluid of blood density within the pleural space. Bronchoscopy may also be diagnostic for blood or clots within the bronchial tree, however can be hazardous, with risk of precipitating further bleeding if clots are extracted. Other imaging modalities such as magnetic resonance imaging are less useful in the emergency setting.

Management requires immediate resuscitation and definitive care. Initially, the airway requires protection, with endotracheal intubation. A Carlens double-lumen, left-sided endobronchial tube or Fogarty embolectomy catheter may be positioned on the bleeding side. Alternatively, selective intubation of the unaffected side may be performed to prevent aspiration of blood products into the healthy lung [12, 13]. Many patients exsanguinate prior to definitive intervention, or are not suitable candidates for intervention. For those that proceed to definitive intervention, a surgical or endoscopic approach may be used. Definitive surgical management may involve primary or patch repair of the aortic end of the fistula, extra-anatomical bypass grafting or prosthetic graft insertion. The pulmonary end of the fistula may be closed primarily, or managed with partial lobe resection, lobectomy or pneumonectomy [3]. For those patients who survive surgery, postoperative 30-day mortality remains as high as 15.3% in case series [3].

Endovascular stenting is associated with lower mortality and recurrence rates than open surgical repair, with 30-day mortality rate of 5.9% and 11.1% rate of recurrence in a series of 134 cases that were followed up for a mean 17.4 months [14]. The need for postoperative antibiotics is inconclusive, with some clinicians advocating the use of antibiotics for up to 4 weeks to prevent graft infection [15].

## CONCLUSION

In summary, while exceedingly rare, aorto-bronchial and aortoenteric fistula should be considered amongst the differentials for upper gastrointestinal bleeding in patients with *in situ* aortic vascular grafts. These pathologies have high mortality and require urgent identification, resuscitation and timely intervention to offer best chance of survival. Furthermore, a history of gastrointestinal infection such as complicated diverticulitis (as occurred in this case) may be a predisposing factor for vascular graft infection and subsequent aorto-bronchial fistula formation.
